# Dental Characteristics of Different Types of Cleft and Non-cleft Individuals

**DOI:** 10.3389/fcell.2020.00789

**Published:** 2020-08-25

**Authors:** Mohammad Khursheed Alam, Ahmed Ali Alfawzan

**Affiliations:** ^1^Orthodontic Division, Department of Preventive Dental Science, College of Dentistry, Jouf University, Sakaka, Saudi Arabia; ^2^Department of Preventive Dentistry, College of Dentistry in Ar Rass, Qassim University, Ar Rass, Saudi Arabia

**Keywords:** non-syndromic cleft lip and palate, bilateral cleft lip and palate, unilateral cleft lip and palate, dental characteristics, overjet, overbite, incisal display

## Abstract

**Objective:**

The objective of this study was to compare the novel artificial intelligence (A.I.)-driven lateral cephalometric (Late. Ceph.) analysis of 14 different dental characteristics (DC) among different types of cleft lip and palate (CLP) and non-cleft (NC) individuals.

**Materials and Methods:**

A retrospective study was conducted on 123 individuals [31 = NC, 29 = BCLP (bilateral cleft lip and palate), 41 = UCLP (unilateral cleft lip and palate), 9 = UCLA (unilateral cleft lip and alveolus), and 13 = UCL (unilateral cleft lip)] with an average age of 14.77 years. Demographic details were gathered from the clinical records. A novel artificial intelligence-driven Webceph software has been used for the Late. Ceph. analysis. A total of 14 different types of angular and linear DC measurements were analyzed and compared among groups. Two-way ANOVA and multiple-comparison statistics tests were applied to see the differences between gender and among different types of CLP versus NC subjects.

**Results:**

Of the 14 DC tested, no significant gender disparities were found (*p* > 0.05). In relation to different types of CLP versus NC subjects, 8 over 14 DC were statistically significant (*p* < 001 to *p* = 0.03). Six other DC variables show insignificant (*p* > 0.05) noteworthy alterations in relation to type of CLP.

**Conclusion:**

Based on the results, type of CLP revealed significantly altered DC compared to NC. Among different types of CLP, BCLP exhibited a maximum alteration in different DC.

## Introduction

Any deformations (anatomical or chromosomal) that start during pregnancy and their belongings identified after birth are considered intrinsic oddities (Sekhon et al., 2011). Among them, cleft lip and palate (CLP) is one of the most widely recognized and major inherent craniofacial peculiarities in humans, brought about by strange facial development during embryogenesis that presents during childbirth and portrayed by halfway or complete clefting of the upper lip, with or without clefting of the alveolar edge or the hard or soft palate (Erverdi and Motro, 2015). Cleft can happen along with CLP or independently like a detached cleft lip and or isolated cleft palate. The point when cleft lip and palate emerge together is named as CLP. The highlights of CLP went from the least serious to the most extreme structure with a unilateral or bilateral manner. CLP can be syndromic or non-syndromic. Clinically, when CLP shows up with other deformities (normally at least two or more), for an inconspicuous example, it is delegated syndromic CLP. In the event that it shows up as a secluded deformity or if the disorder cannot be recognized, the term non-syndromic CLP (NSCLP) is utilized (Kohli and Kohli, 2012).

The etiology of CLP is still controversial. According to previous studies, it is to be thought that both genetic and environmental factors are responsible for CLP (Alam et al., 2012; Berkowitz, 2013; Haque et al., 2015; Haque and Alam, 2015a, c). Studies of the etiology of non-syndromic clefts pivot on candidate genes associated with craniofacial development, genes influenced by environmental teratogens or deficiencies, and genes associated with syndromic clefts (Murray, 2002; Haque et al., 2015). CLP shows significant heterogeneity among different ethnic groups.

Numerous strategies for the evaluation of the craniofacial characteristics, dental relationship, and maxillary morphometry measurement of CLP individuals have been depicted already (Alam et al., 2008, 2013, 2019; Kajii et al., 2013; Asif et al., 2016; Arshad et al., 2017a, b, 2018; Haque et al., 2017a, b, 2018). The result of the craniofacial characteristics of CLP can be evaluated from multifacets of factors, for example, dental relationship (Haque et al., 2018), cephalogram (Alam et al., 2013, 2019; Wu et al., 2013; Batwa et al., 2018; Alam and Alfawzan, 2020), cone-beam computed tomography (Parveen et al., 2018), and maxillary morphometry (Haque et al., 2020). Oral clefts show an assortment of clinical inconsistencies (Batwa et al., 2018). Lee et al. (2020) and Kunz et al. (2020) uncovered artificial intelligence (A.I.) into dentistry, particularly in orthodontics ready to break down obscure Late. Ceph. at nearly a similar quality level as the ongoing highest-quality level estimated by a calibrated specialist. Lee et al. (2020) used A.I.-driven profound convolutional neural system-based assessment of Late. Ceph. for the sign of orthognathic surgery cases of differential determination and discovered 95.6% exactness.

This first-in-human study in a Saudi Arabian population, among different types of NSCLP and NC individuals, is yet to be investigated in regard to different dental characteristics (DC). Hence, in the present study an attempt is made to contribute a novel A.I.-driven analysis of different DC in multiple types of NSCLP and to compare the findings with gender- and age-matched NC individuals. Hence, this study aimed to investigate (1) how the DC are different among gender, (2) how the disparities in DC exist in multiple types of NSCLP and NC individuals, and (3) how the disparities exist in gender times multiple types of NSCLP and NC individuals. The hypothesis of this study is as follows: types of DC are different in relation to gender, type of NSCLP, and NC subjects.

## Materials and Methods

All the records (clinical and demographic details, X-rays) were collected from Saudi Board of dental residents. The research protocol was arranged by one calibrated orthodontist, and the data was stored. The research protocol was presented to the Ethical Committee of Al rass Dental Research Center, Qassim University. Full Ethical approval was obtained with the code #: DRC/009FA/20. The following inclusion and exclusion criteria are followed, non-syndromic cleft subjects with good-quality x-ray images. There was no history of craniofacial surgical treatment besides cleft lip and palate surgery. No orthodontic treatment was done. A match with healthy control without any craniofacial deformity was found.

**FIGURE 1:**
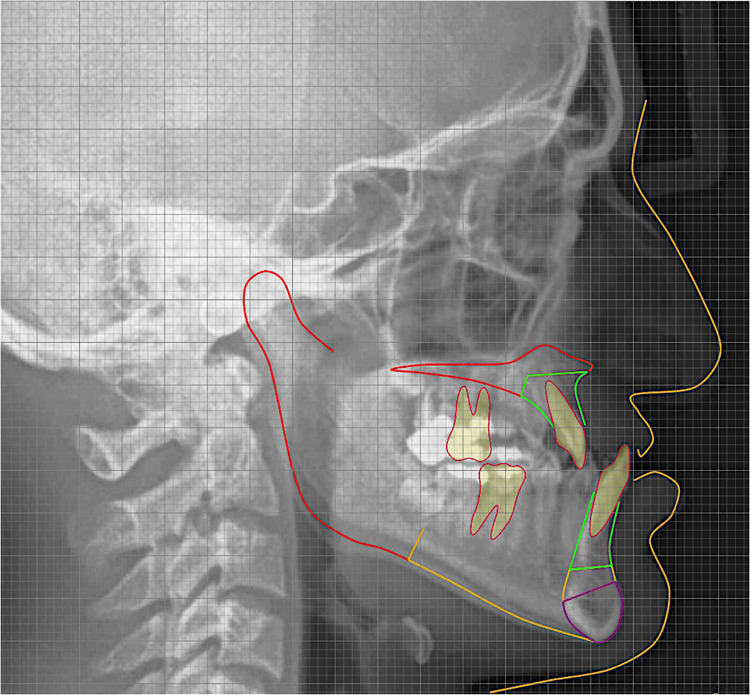
Artificial intelligence-driven lateral cephalometric analysis.

Digital Late. Ceph. X-rays were used to investigate 14 different DC of 123 NC and cleft subjects based on convenient sampling following inclusion and exclusion criteria. Among them, 31 NC subjects and 92 cleft subjects [29 had BCLP (bilateral cleft lip and palate), 41 had UCLP (unilateral cleft lip and palate), 9 had UCLA (unilateral cleft lip and alveolus), and 13 had UCL (unilateral cleft lip)]. According to gender, male = 14 NC + 19 BCLP + 26 UCLP + 3 UCLA + 7 UCL and female = 17 NC + 10 BCLP + 15 UCLP + 6 UCLA + 6 UCL. Ages of the subjects were 13.29 ± 3.52 NC, 14.07 ± 4.73 BCLP, 14.32 ± 4.46 UCLP, 12.78 ± 4.09 UCLA, and 13.31 ± 4.46 UCL. In this retrospective study, clinical and radiographic details were used. Fourteen (14) different DC were measured by one examiner using automated A.I.-driven Webceph software (South Korea). The angular and linear measurements used in this study are detailed in Table 1 and Figure 1.

**TABLE 1:** Dental characteristic measured in NSCLP and NC individuals.

**Variables**	**Short form**	**Details**
Overjet	OJ	Extent of horizontal (anterior-posterior) overlap of the maxillary central incisors over the mandibular central incisors
Overbite	OB	Extent of vertical (superior-inferior) overlap of the maxillary central incisors over the mandibular central incisors
Upper 1 to Frankfort horizontal plane	U1 to FH	Angle between long axis of upper incisor and Frankfort horizontal plane
Upper 1 to sella-nasion plane	U1 to SN	Angle between long axis of upper incisor and sella-nasion plane
Upper 1 to upper occlusal plane	U1 to UOP	Angle between long axis of upper incisor and upper occlusal plane
Incisor mandibular plane angle	IMPA	Angle between long axis of lower incisor and mandibular plane angle
Lower 1 to lower occlusal plane	L1 to LOP	Angle between long axis of lower incisor and lower occlusal plane
Inter-incisor angle	IIA	Angle between long axis of upper and lower incisor
Cant of occlusal plane	COP	Occlusal plane to FH plane
Upper 1 to nasion and point A	U1 to NA (mm)	Distance from upper incisor edge to nasion to point A plane
Upper 1 to nasion and point A	U1 to NA (degree)	Angle between long axis of upper incisor and nasion to point A plane
Lower 1 to nasion and point B	L1 to NB (mm)	Distance from lower incisor edge to nasion to point B plane
Lower 1 to nasion and point B	L1 to NB (degree)	Angle between long axis of lower incisor and nasion to point B plane
Upper incisal display	UID	Maxillary incisal display is one of the most important attributes of smile esthetics. The maximum distance from the lowest point of upper lip to the incisal edge of any of the upper incisor

### Statistical Analyses

To survey the estimation mistake, 20 Late. Ceph. cases were arbitrarily chosen and the means of A.I.-driven investigation were rehashed by one analyst following 2 weeks of first examination. Intra-class correlation coefficients were performed to evaluate the unwavering quality for the two arrangements of estimations. The estimations of coefficients of unwavering quality were seen as more prominent than 0.95 and 0.91 for all linear and angular variables, respectively. Data were analyzed in SPSS (SPSS Inc., Chicago, IL, United States). The Kolmogorov–Smirnov test was utilized to check the normality of the estimations. A two-way ANOVA examination was utilized for gender orientation, types of cleft and gender^∗^types of cleft. A **p**-esteem < 0.05 was considered as significant statistically.

## Results

**FIGURE 2:**
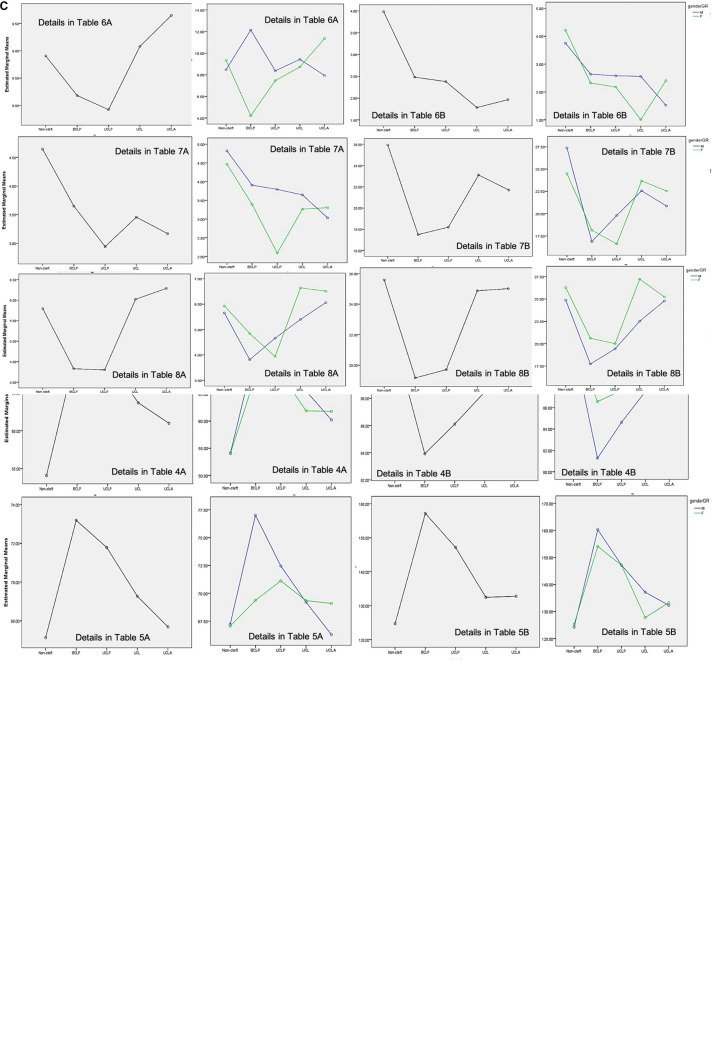
**(A–C)** Profile plot of the estimated marginal means of types of cleft and gender^∗^types of cleft.

Tables 2–8 show the details of the analyzed results of 14 different DC among gender, types of cleft and gender^∗^types of cleft. Figures 2A–C show the profile plot of estimated marginal means of types of cleft and gender^∗^types of cleft.

**TABLE 2:** Dental characteristics – **(A)** Overjet and **(B)** Overbite: Gender, types of cleft and gender times types of cleft two-way ANOVA analysis results.

**Gender**	**Type**	**Mean**	**SD**	**Cleft Type**	**Mean**	**Multiple comparison**			**SE**	***p*-value**	**95% CI**	
											**Lower bound**	**Upper bound**
**(A) Overjet**												
Male	NC	4.449	2.016	NC	4.429	NC	vs	BCLP	11.573*	1.144	0.000	8.299
	BCLP	–5.801	5.104	BCLP	–7.144		vs	UCLP	8.064*	0.992	0.000	5.224
	UCLP	–4.098	5.299	UCLP	–3.635		vs	UCL	4.359*	1.378	0.020	0.413
	UCL	0.021	5.147	UCL	0.071		vs	UCLA	4.548	1.650	0.068	–0.176
	UCLA	–0.523	4.547	UCLA	–0.118	BCLP	vs	UCLP	−3.509*	1.080	0.015	–6.602
	Total	–2.153	5.960				vs	UCL	−7.215*	1.443	0.000	–11.346
Female	NC	4.410	2.602				vs	UCLA	−7.026*	1.704	0.001	–11.905
	BCLP	–8.486	5.485			UCLP	vs	UCL	–3.706	1.326	0.061	–7.502
	UCLP	–3.173	3.342				vs	UCLA	–3.517	1.606	0.306	–8.116
	UCL	0.120	1.266			UCL	vs	UCLA	0.189	1.870	1.000	–5.164
	UCLA	0.287	2.725									
	Total	–1.015	5.506									
										
Total	NC	4.427	2.317			***p*-value**	**PES**					
										
	BCLP	–6.542	5.256		Gender	0.846	0.000					
	UCLP	–3.646	4.423		Cleft Type	0.000	0.512					
	UCL	0.067	3.730		Gender * Cleft Type	0.566	0.026					
										
	UCLA	–0.253	3.866									
	Total	–1.653	5.770									
**(B) Overbite**												
Male	NC	1.237	2.441	NC	1.571	NC	vs	BCLP	0.764	1.000	–2.271	2.107
	BCLP	1.638	3.978	BCLP	1.653		vs	UCLP	0.663	1.000	–1.921	1.876
	UCLP	1.643	3.147	UCLP	1.593		vs	UCL	0.921	1.000	–1.170	4.104
	UCL	1.159	1.650	UCL	0.104		vs	UCLA	1.103	1.000	–3.022	3.292
	UCLA	1.470	1.972	UCLA	1.437	BCLP	vs	UCLP	0.722	1.000	–2.008	2.127
	Total	1.495	3.045				vs	UCL	0.964	1.000	–1.212	4.310
Female	NC	1.905	1.240				vs	UCLA	1.139	1.000	–3.045	3.478
	BCLP	1.669	3.872			UCLP	vs	UCL	0.886	0.957	–1.048	4.027
	UCLP	1.544	2.381				vs	UCLA	1.074	1.000	–2.917	3.231
	UCL	–0.950	0.856			UCL	vs	UCLA	1.250	1.000	–4.910	2.246
	UCLA	1.403	1.270									
	Total	1.391	2.309									
										
Total	NC	1.604	1.875			***p*-value**	**PES**					
										
	BCLP	1.646	3.879		Gender	0.607	0.002					
	UCLP	1.595	2.766		Cleft Type	0.510	0.028					
	UCL	0.185	1.692		Gender * Cleft Type	0.683	0.020					
										
	UCLA	1.448	1.684									
	Total	1.449	2.736									

*SD, standard deviation; MD, mean difference; SE, standard error; CI, confidence interval; and PES, partial eta square.*

**TABLE 3:** Dental characteristics – **(A)** U1 to FH and **(B)** U1 to SN: Gender, types of cleft and gender times types of cleft two-way ANOVA analysis results.

**Gender**	**Type**	**Mean**	**SD**	**Cleft type**	**Mean**	**Multiple comparison**			**SE**	***p*-value**	**95% CI**	
											**Lower bound**	**Upper bound**
**(A) U1 to FH**												
Male	NC	116.074	8.465	NC	115.416	NC	vs	BCLP	2.988	0.000	17.360	34.473
	BCLP	86.171	11.990	BCLP	89.500		vs	UCLP	2.592	0.000	9.285	24.128
	UCLP	99.056	14.532	UCLP	98.710		vs	UCL	3.601	0.381	–2.753	17.867
	UCL	103.914	12.800	UCL	107.860		vs	UCLA	4.311	0.470	–3.684	21.001
	UCLA	107.443	5.413	UCLA	106.758	BCLP	vs	UCLP	2.823	0.015	–17.292	–1.128
	Total	99.809	15.927				vs	UCL	3.770	0.000	–29.155	–7.564
Female	NC	114.759	4.750				vs	UCLA	4.453	0.002	–30.009	–4.508
	BCLP	92.829	13.762			UCLP	vs	UCL	3.465	0.094	–19.070	0.771
	UCLP	98.365	9.516				vs	UCLA	4.198	0.577	–20.067	3.971
	UCL	111.805	10.308			UCL	vs	UCLA	4.886	1.000	–12.887	15.090
	UCLA	106.073	10.698									
	Total	104.627	12.382									
										
Total	NC	115.353	6.597			***p-*value**	**PES**					
										
	BCLP	88.008	12.618		Gender	0.352	0.008					
	UCLP	98.719	12.195		Cleft Type	0.000	0.432					
	UCL	107.556	11.956		Gender * Cleft Type	0.482	0.030					
										
	UCLA	106.987	6.885									
	Total	101.925	14.620									
**(B) U1 to SN**												
Male	NC	106.671	8.479	NC	105.731	NC	vs	BCLP	3.172	0.000	17.509	35.673
	BCLP	76.177	13.008	BCLP	79.140		vs	UCLP	2.751	0.000	8.487	24.242
	UCLP	90.420	15.290	UCLP	89.367		vs	UCL	3.822	0.945	–4.498	17.389
	UCL	95.234	13.826	UCL	99.285		vs	UCLA	4.576	0.987	–5.482	20.719
	UCLA	99.395	6.536	UCLA	98.113	BCLP	vs	UCLP	2.996	0.009	–18.805	–1.648
	Total	90.651	16.695				vs	UCL	4.002	0.000	–31.604	–8.687
Female	NC	104.792	5.593				vs	UCLA	4.727	0.001	–32.506	–5.439
	BCLP	82.104	15.417			UCLP	vs	UCL	3.678	0.081	–20.448	0.611
	UCLP	88.314	9.676				vs	UCLA	4.455	0.521	–21.502	4.011
	UCL	103.337	10.000			UCL	vs	UCLA	5.186	1.000	–13.674	16.020
	UCLA	96.830	10.398									
	Total	94.724	12.985									
										
Total	NC	105.640	6.982			***p*-value**	**PES**					
										
	BCLP	77.812	13.695		Gender	0.556	0.003					
	UCLP	89.393	12.748		Cleft Type	0.000	0.416					
	UCL	98.974	12.447		Gender * Cleft Type	0.456	0.031					
										
	UCLA	98.540	7.441									
	Total	92.439	15.256									

*SD, standard deviation; MD, mean difference; SE, standard error; CI, confidence interval; and PES, partial eta square.*

**TABLE 4:** Dental characteristics – **(A)** U1 to UOP and **(B)** IMPA: Gender, types of cleft and gender times types of cleft two-way ANOVA analysis results.

**Gender**	**Type**	**Mean**	**SD**	**Cleft type**	**Mean**	**Multiple comparison**			**SE**	***p*-value**	**95% CI**	
											**Lower bound**	**Upper bound**
**(A) U1 to UOP**												
Male	NC	54.119	6.073	NC	54.075	NC	vs	BCLP	2.658	0.000	–24.426	–9.207
	BCLP	73.341	12.229	BCLP	70.891		vs	UCLP	2.305	0.000	–21.969	–8.768
	UCLP	70.295	12.922	UCLP	69.443		vs	UCL	3.202	0.033	–18.783	–0.444
	UCL	65.503	7.232	UCL	63.688		vs	UCLA	3.834	0.740	–17.890	4.063
	UCLA	60.197	3.379	UCLA	60.988	BCLP	vs	UCLP	2.510	1.000	–5.740	8.636
	Total	66.576	12.636				vs	UCL	3.353	0.338	–2.398	16.804
Female	NC	54.030	4.391				vs	UCLA	3.961	0.138	–1.437	21.243
	BCLP	68.441	11.177			UCLP	vs	UCL	3.081	0.644	–3.067	14.578
	UCLP	68.592	10.414				vs	UCLA	3.733	0.254	–2.234	19.144
	UCL	61.873	3.587			UCL	vs	UCLA	4.345	1.000	–9.741	15.140
	UCLA	61.780	5.103									
	Total	62.860	10.280									
										
Total	NC	54.070	5.125			***p*-value**	**PES**					
										
	BCLP	71.990	11.959		Gender	0.412	0.006					
	UCLP	69.464	11.651		Cleft Type	0.000	0.338					
	UCL	63.828	5.921		Gender * Cleft Type	0.878	0.010					
										
	UCLA	60.724	3.778									
	Total	64.945	11.761									
**(B) IMPA**												
Male	NC	91.971	8.365	NC	92.173	NC	vs	BCLP	2.051	0.001	2.380	14.127
	BCLP	81.274	8.759	BCLP	83.920		vs	UCLP	1.779	0.009	0.969	11.159
	UCLP	84.625	8.473	UCLP	86.109		vs	UCL	2.472	1.000	–3.376	10.779
	UCL	87.520	4.118	UCL	88.472		vs	UCLA	2.959	1.000	–5.819	11.126
	UCLA	89.982	4.400	UCLA	89.519	BCLP	vs	UCLP	1.938	1.000	–7.737	3.359
	Total	85.855	8.741				vs	UCL	2.588	0.813	–11.963	2.859
Female	NC	92.374	6.227				vs	UCLA	3.057	0.696	–14.352	3.153
	BCLP	86.565	2.899			UCLP	vs	UCL	2.378	1.000	–9.173	4.447
	UCLP	87.593	7.980				vs	UCLA	2.882	1.000	–11.661	4.840
	UCL	89.423	7.148			UCL	vs	UCLA	3.354	1.000	–10.650	8.555
	UCLA	89.057	5.356									
	Total	89.230	6.841									
										
Total	NC	92.192	7.144			***p*-value**	**PES**					
										
	BCLP	82.734	7.918		Gender	0.242	0.012					
	UCLP	86.073	8.270		Cleft Type	0.001	0.147					
	UCL	88.398	5.545		Gender * Cleft Type	0.755	0.016					
										
	UCLA	89.673	4.414									
	Total	87.337	8.109									

*SD, standard deviation; MD, mean difference; SE, standard error; CI, confidence interval; and PES, partial eta square.*

**TABLE 5:** Dental characteristics – **(A)** L1 to LOP and **(B)** inter-incisor angle: Gender, types of cleft and gender times types of cleft two-way ANOVA analysis results.

**Gender**	**Type**	**Mean**	**SD**	**Cleft type**	**Mean**	**Multiple comparison**			**SE**	***p*-value**	**95% CI**	
											**Lower bound**	**Upper bound**
**(A) L1 to LOP**												
Male	NC	67.216	7.982	NC	67.133	NC	vs	BCLP	1.991	0.029	–11.757	–0.355
	BCLP	77.005	8.648	BCLP	73.189		vs	UCLP	1.727	0.081	–9.599	0.292
	UCLP	72.454	6.708	UCLP	71.786		vs	UCL	2.399	1.000	–9.007	4.733
	UCL	69.199	5.452	UCL	69.270		vs	UCLA	2.872	1.000	–8.782	7.666
	UCLA	66.292	3.959	UCLA	67.691	BCLP	vs	UCLP	1.881	1.000	–3.982	6.788
	Total	71.910	8.208				vs	UCL	2.512	1.000	–3.274	11.112
Female	NC	67.050	7.733				vs	UCLA	2.967	0.665	–2.998	13.994
	BCLP	69.374	6.580			UCLP	vs	UCL	2.309	1.000	–4.094	9.126
	UCLP	71.119	7.269				vs	UCLA	2.797	1.000	–3.913	12.104
	UCL	69.342	3.906			UCL	vs	UCLA	3.255	1.000	–7.741	10.900
	UCLA	69.090	6.946									
	Total	69.269	6.989									
										
Total	NC	67.125	7.714			***p*-value**	**PES**					
										
	BCLP	74.900	8.734		Gender	0.438	0.005					
	UCLP	71.803	6.932		Cleft Type	0.017	0.100					
	UCL	69.265	4.607		Gender * Cleft Type	0.271	0.044					
										
	UCLA	67.224	4.880									
	Total	70.751	7.778									
**(B)Inter-incisor angle**												
Male	NC	124.194	13.399	NC	124.704	NC	vs	BCLP	3.828	0.000	–43.443	–21.523
	BCLP	160.287	13.646	BCLP	157.186		vs	UCLP	3.320	0.000	–31.953	–12.939
	UCLP	147.191	19.669	UCLP	147.149		vs	UCL	4.613	0.951	–20.971	5.443
	UCL	137.156	14.119	UCL	132.468		vs	UCLA	3.828	0.000	21.523	43.443
	UCLA	132.308	4.941	UCLA	132.786	BCLP	vs	UCLP	3.616	0.064	–0.316	20.390
	Total	144.198	20.123				vs	UCL	4.830	0.000	10.890	38.547
Female	NC	125.214	10.023				vs	UCLA	5.705	0.000	8.067	40.734
	BCLP	154.086	13.486			UCLP	vs	UCL	4.438	0.013	1.974	27.389
	UCLP	147.108	13.318				vs	UCLA	5.377	0.087	–1.032	29.759
	UCL	127.780	5.060			UCL	vs	UCLA	6.258	1.000	–18.236	17.601
	UCLA	133.263	13.700									
	Total	138.332	16.224									
										
Total	NC	124.753	11.474			***p*-value**	**PES**					
										
	BCLP	158.576	13.654		Gender	0.373	0.007					
	UCLP	147.150	16.664		Cleft Type	0.000	0.441					
	UCL	132.828	11.576		Gender * Cleft Type	0.721	0.018					
										
	UCLA	132.627	7.900									
	Total	141.623	18.671									

*SD, standard deviation; MD, mean difference; SE, standard error; CI, confidence interval; and PES, partial eta square.*

**TABLE 6:** Dental characteristics – **(A)** Cant of occlusal plane and **(B)** Upper incisal display: Gender, types of cleft and gender times types of cleft two-way ANOVA analysis results.

**Gender**	**Type**	**Mean**	**SD**	**Cleft type**	**Mean**	**Multiple comparison**			**SE**	***p*-value**	**95% CI**	
											**Lower bound**	**Upper bound**
**(A) Cant of occlusal plane**												
Male	NC	8.480	3.892	NC	124.704	NC	vs	BCLP	1.433	1.000	–3.378	4.829
	BCLP	12.146	4.315	BCLP	157.186		vs	UCLP	1.243	1.000	–2.576	4.543
	UCLP	8.377	5.113	UCLP	147.149		vs	UCL	1.727	1.000	–5.118	4.771
	UCL	9.430	5.911	UCL	132.468		vs	UCLA	2.067	1.000	–6.661	5.178
	UCLA	7.943	3.873	UCLA	132.786	BCLP	vs	UCLP	1.354	1.000	–3.618	4.134
	Total	9.614	4.818				vs	UCL	1.808	1.000	–6.076	4.278
Female	NC	9.334	3.494				vs	UCLA	2.136	1.000	–7.582	4.648
	BCLP	4.216	7.823			UCLP	vs	UCL	1.662	1.000	–5.914	3.601
	UCLP	7.470	6.710				vs	UCLA	2.013	1.000	–7.489	4.039
	UCL	8.730	5.553			UCL	vs	UCLA	2.343	1.000	–7.277	6.140
	UCLA	11.353	5.241									
	Total	7.930	5.948									
										
Total	NC	8.948	3.642			***p*-value**	**PES**					
										
	BCLP	9.959	6.451		Gender	0.359	0.007					
	UCLP	7.934	5.888		Cleft Type	0.857	0.012					
	UCL	9.107	5.518		Gender * Cleft Type	0.018	0.099					
										
	UCLA	9.080	4.376									
	Total	8.875	5.387									
**(B)Upper incisal display**												
Male	NC	3.750	3.093	NC	3.982	NC	vs	BCLP	0.792	0.607	–0.767	3.770
	BCLP	2.640	3.650	BCLP	2.480		vs	UCLP	0.687	0.215	–0.365	3.570
	UCLP	2.579	2.497	UCLP	2.379		vs	UCL	0.955	0.232	–0.536	4.932
	UCL	2.560	2.290	UCL	1.784		vs	UCLA	1.143	0.803	–1.255	5.290
	UCLA	1.525	2.960	UCLA	1.964	BCLP	vs	UCLP	0.749	1.000	–2.042	2.244
	Total	2.741	3.007				vs	UCL	1.000	1.000	–2.166	3.559
Female	NC	4.214	2.099				vs	UCLA	1.181	1.000	–2.865	3.897
	BCLP	2.321	3.649			UCLP	vs	UCL	0.919	1.000	–2.035	3.226
	UCLP	2.180	2.806				vs	UCLA	1.113	1.000	–2.772	3.602
	UCL	1.008	1.927			UCL	vs	UCLA	1.296	1.000	–3.889	3.529
	UCLA	2.403	2.680									
	Total	2.723	2.778									
										
Total	NC	4.004	2.560			***p*-value**	**PES**					
										
	BCLP	2.552	3.587		Gender	0.770	0.001					
	UCLP	2.384	2.627		Cleft Type	0.081	0.070					
	UCL	1.844	2.195		Gender * Cleft Type	0.833	0.013					
										
	UCLA	1.818	2.732									
	Total	2.733	2.897									

*SD, standard deviation; MD, mean difference; SE, standard error; CI, confidence interval; and PES, partial eta square.*

**TABLE 7:** Dental characteristics – **(A)** U1 to NA (mm) and **(B)** U1 to NA (degree): Gender, types of cleft and gender times types of cleft two-way ANOVA analysis results.

**Gender**	**Type**	**Mean**	**SD**	**Cleft type**	**Mean**	**Multiple comparison**			**SE**	***p*-value**	**95% CI**	
											**Lower bound**	**Upper bound**
**(A) U1 to NA (mm)**												
Male	NC	4.823	2.557	NC	4.645	NC	vs	BCLP	0.699	1.000	–1.007	2.996
	BCLP	3.907	2.706	BCLP	3.650		vs	UCLP	0.606	0.059	–0.033	3.439
	UCLP	3.792	3.049	UCLP	2.942		vs	UCL	0.842	1.000	–1.223	3.600
	UCL	3.646	2.417	UCL	3.456		vs	UCLA	1.008	1.000	–1.410	4.365
	UCLA	3.032	2.393	UCLA	3.167	BCLP	vs	UCLP	0.660	1.000	–1.183	2.599
	Total	3.955	2.706				vs	UCL	0.882	1.000	–2.332	2.719
Female	NC	4.466	1.927				vs	UCLA	1.042	1.000	–2.501	3.465
	BCLP	3.393	3.429			UCLP	vs	UCL	0.811	1.000	–2.835	1.806
	UCLP	2.092	1.715				vs	UCLA	0.982	1.000	–3.037	2.586
	UCL	3.267	2.428			UCL	vs	UCLA	1.143	1.000	–2.984	3.561
	UCLA	3.303	3.260									
	Total	3.230	2.381									
										
Total	NC	4.627	2.201			***p*-value**	**PES**					
										
	BCLP	3.765	2.868		Gender	0.340	0.008					
	UCLP	2.963	2.605		Cleft Type	0.091	0.068					
	UCL	3.471	2.328		Gender * Cleft Type	0.729	0.018					
										
	UCLA	3.122	2.501									
	Total	3.637	2.584									
**(B)U1 to NA (degree)**												
Male	NC	27.376	8.148	NC	25.938	NC	vs	BCLP	1.584	0.000	3.903	12.974
	BCLP	16.857	4.241	BCLP	17.499		vs	UCLP	1.374	0.000	3.807	11.675
	UCLP	19.793	5.928	UCLP	18.197		vs	UCL	1.909	1.000	–2.642	8.289
	UCL	22.557	5.638	UCL	23.114		vs	UCLA	2.285	0.659	–2.300	10.785
	UCLA	20.850	5.838	UCLA	21.695	BCLP	vs	UCLP	1.496	1.000	–4.982	3.586
	Total	20.810	6.925				vs	UCL	1.999	0.058	–11.338	.107
Female	NC	24.500	3.660				vs	UCLA	2.361	0.782	–10.955	2.563
	BCLP	18.141	5.246			UCLP	vs	UCL	1.837	0.085	–10.176	.341
	UCLP	16.601	5.426				vs	UCLA	2.225	1.000	–9.869	2.873
	UCL	23.672	9.276			UCL	vs	UCLA	2.590	1.000	–5.996	8.834
	UCLA	22.540	5.545									
	Total	20.431	6.371									
										
Total	NC	25.799	6.167			***p-*value**	**PES**					
										
	BCLP	17.211	4.480		Gender	0.755	0.001					
	UCLP	18.236	5.845		Cleft Type	0.000	0.274					
	UCL	23.072	7.217		Gender * Cleft Type	0.417	0.034					
										
	UCLA	21.413	5.450									
	Total	20.644	6.663									

*SD, standard deviation; MD, mean difference; SE, standard error; CI, confidence interval; and PES, partial eta square.*

**TABLE 8:** Dental characteristics – **(A)** L1 to NB (mm) and **(B)** L1 to NB (degree): Gender, types of cleft and gender times types of cleft two-way ANOVA analysis results.

**Gender**	**Type**	**Mean**	**SD**	**Cleft type**	**Mean**	**Multiple comparison**			**SE**	***p*-value**	**95% CI**	
											**Lower bound**	**Upper bound**
**(A) L1 to NB (mm)**												
Male	NC	5.654	3.036	NC	25.938	NC	vs	BCLP	0.721	0.447	–0.601	3.530
	BCLP	3.811	2.436	BCLP	17.499		vs	UCLP	0.626	0.187	–0.299	3.285
	UCLP	4.660	2.710	UCLP	18.197		vs	UCL	0.869	1.000	–2.716	2.262
	UCL	5.397	1.772	UCL	23.114		vs	UCLA	1.041	1.000	–3.474	2.486
	UCLA	6.062	1.504	UCLA	21.695	BCLP	vs	UCLP	0.681	1.000	–1.922	1.980
	Total	4.800	2.597				vs	UCL	0.910	0.658	–4.297	0.915
Female	NC	5.930	3.053				vs	UCLA	1.075	0.712	–5.036	1.120
	BCLP	4.844	2.575			UCLP	vs	UCL	0.836	0.421	–4.115	0.675
	UCLP	3.938	2.126				vs	UCLA	1.013	0.524	–4.889	0.914
	UCL	6.640	2.782			UCL	vs	UCLA	1.179	1.000	–3.644	3.110
	UCLA	6.510	4.526									
	Total	5.142	2.817									
										
Total	NC	5.805	2.998			***p*-value**	**PES**					
										
	BCLP	4.096	2.473		Gender	0.431	0.005					
	UCLP	4.308	2.440		Cleft Type	0.030	0.090					
	UCL	5.971	2.283		Gender * Cleft Type	0.666	0.021					
										
	UCLA	6.211	2.566									
	Total	4.950	2.690									
**(B)L1 to NB (degree)**												
Male	NC	24.875	6.460	NC	25.582	NC	vs	BCLP	1.993	0.017	0.708	12.120
	BCLP	17.726	7.604	BCLP	19.168		vs	UCLP	1.729	0.009	0.920	10.819
	UCLP	19.421	8.771	UCLP	19.712		vs	UCL	2.401	1.000	–6.173	7.578
	UCL	22.524	4.887	UCL	24.880		vs	UCLA	2.875	1.000	–7.664	8.798
	UCLA	24.787	4.940	UCLA	25.015	BCLP	vs	UCLP	1.882	1.000	–5.934	4.846
	Total	20.793	7.755				vs	UCL	2.514	0.250	–12.911	1.488
Female	NC	26.289	6.619				vs	UCLA	2.970	0.514	–14.350	2.656
	BCLP	20.610	5.193			UCLP	vs	UCL	2.311	0.273	–11.783	1.448
	UCLP	20.004	7.808				vs	UCLA	2.799	0.607	–13.318	2.712
	UCL	27.235	6.745			UCL	vs	UCLA	3.258	1.000	–9.464	9.193
	UCLA	25.243	8.616									
	Total	23.167	7.466									
										
Total	NC	25.650	6.478			***p*-value**	**PES**					
										
	BCLP	18.522	7.054		Gender	0.210	0.014					
	UCLP	19.705	8.216		Cleft Type	0.002	0.141					
	UCL	24.698	6.072		Gender * Cleft Type	0.905	0.009					
										
	UCLA	24.939	5.820									
	Total	21.835	7.690									

*SD, standard deviation; MD, mean difference; SE, standard error; CI, confidence interval; and PES, partial eta square.*

In Table 2A, overjet DC is presented, which shows no significant gender disparities and highly significant disparities among NC and different types of clefts (BCLP *p* < 0.001, UCLP *p* < 0.001 and UCL, *p* = 0.020). UCLP *p* = 0.015, UCL *p* < 0.001, and UCLA, *p* = 0.001, showed a significant difference in comparison with BCLP. In relation to overbite DC, no significant disparities were observed (Table 2B).

Tables 3A,B shows U1 to FH and U1 to SN DC with no significant gender disparities and highly significant disparities among NC and different types of clefts (BCLP *p* < 0.001 and UCLP *p* < 0.001) in comparison with NC. UCLP *p* = 0.015, UCL *p* < 0.001, and UCLA, *p* = 0.002, showed significant difference in comparison with BCLP in relation to U1 to FH DC. Moreover, UCLP *p* = 0.009, UCL *p* < 0.001, and UCLA, *p* = 0.001, showed a significant difference in comparison with BCLP in relation to U1 to SN DC.

Tables 4A,B shows U1 to UOP and IMPA DC with significant disparities among NC and different types of clefts (BCLP < 0.001 and *p* = 0.001 and UCLP < 0.001 and *p* = 0.009, respectively).

In relation to L1 to LOP DC, no significant disparities were observed (Table 5A). Table 5B shows inter-incisor angle DC with highly significant disparities among NC and different types of clefts (BCLP < 0.001, UCLP < 0.001, and UCLA < 0.001). UCL < 0.001 and UCLA < 0.001 showed a significant difference in comparison with BCLP. UCL *p* = 0.03 showed a significant difference in comparison with UCLP.

In relation to Cant of occlusal plane, upper incisal display DC, and U1 to NA (mm), no significant disparities were observed (Tables 6A,B, 7A). Table 7B shows U1 to NA (degree) DC with significant disparities among NC and different types of clefts (BCLP *p* = 0.001 and UCLP *p* = 0.009).

Table 8A shows L1 to NB (mm) DC, no significant disparities were observed. L1 to NB (degree) DC show significant disparities among NC and different types of clefts (BCLP *p* = 0.017 and UCLP *p* = 0.009) (Table 8B).

## Discussion

Fourteen (14) distinctive DC of five unique groups of individuals are researched in the present study. As far as we could possibly know, A.I.-driven computerized Late. Ceph. examination in such gatherings and populace is yet to be researched. Irrelevant mistake in the estimations; exact, automated, basic, brisk, savvy, future orthodontic computerized apparatuses; and different types of cleft examples are the novelty of the current examination (Lee et al., 2020; Kunz et al., 2020). The current investigation results may help the clinician in approaching where the impacts of essential CLP medical procedures are on various DC, supporting the restoration procedure in subjects with various sorts of NSCLP in building up a positive administration convention.

Batwa et al. (2018) recommended broadly that analysts in the CLP field should embrace exhaustive activities to survey a wide range of CLP. Longitudinal and extensive examination studies will empower social insurance suppliers to actualize substantial treatment conventions that are suitable for the extraordinary nature and intricacy of the CLP populace. The unilateral complete type of CLP subjects with multiple missing teeth had the significantly smallest overjet (–3.89 ± 2.75 mm) among the three groups (without missing teeth, with only one missing tooth, and with two or more missing teeth). In the current study, overjet in NC = 4.429, BCLP = −7.144, UCLP = −3.635, UCL = 0.071, and UCLA = −0.118 exhibits significant disparities. Maximum alterations are found in the BCLP group. UCLP results almost coincide with the results of Batwa et al. (2018) in which the smallest overjet was found in the unilateral complete type of CLP subjects with multiple missing teeth.

These disparities may be due to multiple-factor relations. When a patient is born with CLP, a number of surgeries take place in the 1st 2 years of life. One study used the presurgical orthopedic feeding plate after birth (Haque and Alam, 2015b); at 3–6 months of age, the patients underwent cheiloplasty (Haque and Alam, 2014), and at 9–18 months of age they underwent palatoplasty (Haque and Alam, 2015c). There was a formation of excessive scar tissues, and the undermining of soft tissue was observed after these surgeries, which may have resulted in maxillary contracture which finally leads to class III malocclusion. Maxillary growth retardation is often observed in patients with repaired unilateral cleft lip and palate (UCLP) (Alam et al., 2008; Kajii et al., 2013). Altered craniofacial morphology was also observed in relation to postnatal treatment factors and congenital factors in the Japanese population (Alam et al., 2013, 2019).

Wu et al. (2013) proposed that further investigations are expected to investigate the skeletal and dental attributes of individuals with CLP in other ethnic gatherings, especially in the Middle Eastern region. They assessed only individuals with unilateral complete CLP among various kinds of CLP. They found various cephalometric characteristics present in Taiwanese people with unilateral complete CLP and found a general decrease in their skeletal vertical measurements and a decrease in the overjet. The current study also revealed a significant alteration in overjet. However, overbite, which determines the vertical dental relationship, shows no significant alterations. Five other DC—L1 to LOP, Cant of occlusal plane, U1 to NA (mm), L1 to NB (mm), and upper incisal display DC—also showed no significant disparities among genders, types of CLP, and NC individuals.

Alam et al. (2019), Alam and Alfawzan (2020) investigated the craniofacial morphology of Japanese UCLP patients and investigated the association with congenital (2019) and postnatal treatment factors (2013). Among congenital factors, gender and DC (U1-SN) showed insignificant disparities, which coincide with the results of the present study. Among postnatal treatment factors, significantly larger U1-SN measurements are found in subjects that underwent preoperative orthopedic treatment with a Hotz plate in comparison with the subjects that underwent no preoperative orthopedic treatment (HOTZ plate) or an active plate. These investigations are researched in UCLP subjects only. The current study compared four types of NSCLP and NC individuals. These disparities may be due to the fact that the management protocol of a patient with cleft is complex and requires a lengthy procedure. The involvement of multi-specialties working in tandem is suggested to bring out physical, psychological, and social rehabilitation. Likewise, maxillary arch constriction (maxillary growth retardation) is a common dental problem of CLP patients, resulting in a concave facial profile (Alam et al., 2019), class III malocclusion (Alam et al., 2013), midfacial growth deficiency (Alam et al., 2013, 2019), and congenitally missing and malformed teeth. Orthodontic anomalies like crowding, rotation, and malposition of teeth are also commonly observed (Haque and Alam, 2015a; Haque et al., 2018; Adetayo et al., 2019). In the current study, maximum alterations in 8 different DC were found to be mostly altered in relation to upper incisors [U1-FH, U1-SN, U1-UOP, IIA, and U1-NA (degree)]. Our results clearly indicate that NSCLP subjects exhibit a class III malocclusion pattern based on investigated multiple DC. Also, the results are more prominent in BCLP individuals.

Batwa et al. (2018) found U1-SN values of 85.04 ± 12.13 and 91.63 ± 10.62 (mean ± SD) in the control and case groups (UCCLP), respectively. Utilizing the mean ± SD values of the two groups, the calculated Cohen’s d and effect-size r were 0.578 and 0.277, respectively. Sample power analysis was done using G^∗^Power software, and the effect size was calculated (Batwa et al., 2018). Based on this, the total sample in the five groups is required to be 103. In each group, 20 or 21 individuals are required with α err prob and power (1-β err prob) values of 0.05 and 80, respectively. Strict inclusion criteria were followed to recruit the data. A good number of BCLP and UCLP samples and age- and sex-matched NC individuals are recruited; however, the sample size of UCLA and UCL is lacking. To draw any strong conclusion in different CLP problems, a genetic investigation may play a beneficial role. Furthermore, genetic/congenital/postnatal treatment factors may influence or alter the shape/growth of the DC. Future studies involving effects of genetic/congenital/postnatal treatment factors along with a greater number of samples may be beneficial in drawing a strong conclusion. The current study cannot state whether comparative discoveries may have been obtained from different individuals with numerous sorts of NSCLP. It may be helpful to do this type of two-way ANOVA examination in bunches from different hospitals/clinics. Future investigations with bigger example sizes are justified.

## Conclusion

• The current study investigated 14 different DC. Among 14 different DC, 8 variables showed a significant alteration among different types of NSCLP and NC individuals.
• No significant gender disparities were found in relation to types of different NSCLP and NC individuals.
• Among CLP, BCLP showed maximum alterations in different DC in relation to NC individuals as well as within other types of CLP individuals.

## Data Availability Statement

All datasets presented in this study are included in the article/Supplementary Material.

## Ethics Statement

The studies involving human participants were reviewed and approved by the Ethical Committee of Al Rass Dental Research Center, Qassim University, Code #: DRC/009FA/20. Written informed consent to participate in this study was provided by the participants’ legal guardian/next of kin.

## Author Contributions

All authors listed have made a substantial, direct and intellectual contribution to the work, and approved it for publication.

## Conflict of Interest

The authors declare that the research was conducted in the absence of any commercial or financial relationships that could be construed as a potential conflict of interest.
